# Circumcision Status and Risk of HIV Acquisition during Heterosexual Intercourse for Both Males and Females: A Meta-Analysis

**DOI:** 10.1371/journal.pone.0125436

**Published:** 2015-05-05

**Authors:** Jun hao LEI, Liang ren LIU, Qiang WEI, Shi bing YAN, Lu YANG, Tu run SONG, Hai chao YUAN, Xiao LV, Ping HAN

**Affiliations:** 1 Department of Urology, West China Hospital, Sichuan University, Chengdu, Sichuan, China; 2 Department of Urology, Dujiangyan Medical Center/ the affiliated hospital of Chengdu University, Dujianyan, China; Alberta Provincial Laboratory for Public Health/ University of Alberta, CANADA

## Abstract

In this study, we evaluated if male circumcision was associated with lower HIV acquisition for HIV (−) males and HIV (−) females during normal sexual behavior. We performed a systematic literature search of PubMed, EMBASE, and Cochrane Central Register of Controlled Trials (CENTRAL) databases to identify studies that compared HIV acquisition for the circumcised and uncircumcised groups. The reference lists of the included and excluded studies were also screened. Fifteen studies (4 RCTs and 11 prospective cohort studies) were included, and the related data were extracted and analyzed in a meta-analysis. Our study revealed strong evidence that male circumcision was associated with reduced HIV acquisition for HIV(−) males during sexual intercourse with females [pooled adjusted risk ratio (RR): 0.30, 95% CI 0.24 0.38, *P * < 0.00001] and provided a 70% protective effect. In contrast, no difference was detected in HIV acquisition for HIV (−) females between the circumcised and uncircumcised groups (pooled adjusted RR after sensitivity analysis: 0.68, 95%CI 0.40–1.15, *P* = 0.15). In conclusion, male circumcision could significantly protect males but not females from HIV acquisition at the population level. Male circumcision may serve as an additional approach toward HIV control, in conjunction with other strategies such as HIV counseling and testing, condom promotion, and so on.

## Introduction

The relationship between circumcision status and the prevention of sexually transmitted diseases (STDs) was described initially by Hutchinson almost 150 years ago [1], and its potential protective effect for HIV acquisition was reported in 1986 [2]. Recently, many prospective studies, particularly three large RCTs, showed that male circumcision provided a protective effect for males [3–5]. A systematic review summarized the three trials published in the Cochrane Library in 2013 and reported a pooled RR of 0.46 at 21 and 24 months (95% CI: 0.34–0.62, *P* = 0.0002) [6]. However, the evidence is inadequate. Many longitudinal studies concerning the relationship between circumcision status of HIV (−) males and HIV acquisition from their female partners should also be considered [7–12]. On the other hand, the available evidence concerning the relationship between male circumcision status and HIV acquisition for their HIV (−) female partners is still inconsistent [13–22].

In the current study, we included RCTs and prospective cohort studies to examine whether male circumcision provided a protective effect for males themselves and for their female partners.

## Methods

### Inclusion Criteria and Search Strategy

Studies that met all the following criteria were included: randomized controlled trial or cohort study: a) in which sexual activity was limited to heterosexuality, b) with male circumcision as a study variable or an exposure variable and c) that reported quantitative data reflecting the association of circumcision status and HIV acquisition for either males or females.

We searched for such studies in the PubMed, EMBASE, and the Cochrane Central Register of Controlled Trials (CENTRAL) in the Cochrane Library. PubMed was searched using the following terms: Circumcision, Male [MeSH] or Circumcision [tw] or male circumcision [tw] or circumcise [tw] or Shang Ring [tw] and HIV Infections [MeSH] or HIV [MeSH] or human immunodeficiency virus [tw] or hiv infect [tw] or AIDS [tw] or Acquired Immune Deficiency Syndrome [MeSH] or STD [tw] or sexually transmitted diseases [MeSH]. The other two databases were searched using similar keywords according to their search rules. The reference lists of the included studies, other reviews, and related articles not identified by our electronic searches were also screened for additional possible studies. HIV (+) individuals were identified after testing positive for two types of enzymes in immunoassays of venous blood samples. Discordant enzyme immunoassay results were confirmed by western blot [19]. To minimize the clinical and methodological heterogeneity, we only included RCTs or cohort studies.

## Data Extractions、Quality Assessment and Data Analysis

LY and PH performed study selection and data extraction independently. The items extracted from the 15 eligible studies are shown in Table 1. The effect measures for unadjusted and adjusted RRs were also extracted. The adjusted RRs were preferentially used for the meta-analysis; otherwise, the unadjusted RRs were used. Any disagreements were solved by discussion within the study group.

**Table 1:** Summary table of correlation of male circumcision status and HIV acquisition.

Study/ study design	sites	population	size analyzed in cir./unc. group	follow up duration	proportion with circumcised male	HIV(+) rate in circumcised group	HIV(+) rate in uncircumcised group	Unadjusted RR# (95% CI^&^)	Adjusted RR(95% CI)
**Studies concerning HIV acquisition from female to male**									
Auvert2005 /RCT	South Africa	male of general population	1546/1582	21mons	49%	0.85 per 100 pyo*	2.1 per 100 pyo	0.40 (0.24–0.68)	0.39 (0.23–0.66)○
Bailey2007 /RCT	Kisumu, Kenya	HIV(-) male	1391/1393	24mons	50%	2.1% (1.2–3.0)	4.2% (3.0–5.4)	0.47 (0.28–0.78)	0.41 (0.24–0.70)◎
Gray2007 /RCT	Rakai, Uganda	HIV(-) male	2474/2522	24mons	50%	0.66 per 100 pyo	1.33 per 100 pyo	0.49 (0.28–0.84)	0.49 (0.29–0.81)⊕
Cameron1989 /P. Cohort	Nairobi, Kenya	STD clinic visitors	214/79	№	73%	№	№	0.10 (0.04–0.22)	0.12 (0.04–0.33)♂
Lavreys1999 /P. Cohort	Mombasa,Kenya	HIV(-) male	651/95	20mons	13%	2.50 per 100 pyo	1.8 per 100 pyo	0.42 (0.22–0.81)	№
Wawer1999 /P. Cohort	Rakai, Uganda	Subgroup1(S1): HIV(-) male	409/1635	20mons	20%	1.40 per 100 pyo	№	0.97 (0.36–2.25)	№
	№	Subgroup2(S2): HIV(-) male	346/1961	20mons	15%	0.70 per 100 pyo	№	0.45 (0.09–1.40)	№
Ronaid2000 /P. Cohort	Rakai, Uganda	HIV(-) male	908/4608	49mons	16%	1.10 per 100 pyo	1.8 per 100 pyo	0.61 (0.37–0.97)	0.53(0.33–0.87))⊙
Reynolds2004 /P. Cohort	Pune, India	HIV(-) male	191/2107	12mons	8%	0.70 per 100 pyo	5.50 per 100 pyo	0.13 (0.02–0.47)	0.15 (0.04–0.62)♀
Gray2012/P. Cohort	Rakai, Uganda	HIV(-) male	3198/402	4.79years	89%	0.50 per 100 pyo	1.93per 100 pyo	0.27 (0.16–0.44)	0.27 (0.16–0.45)☆
**Studies concerning HIV acquisition from male to female**									
Wawer2009 /RCT	Rakai, Uganda	Female with HIV(+) partners	92/67	24mons	58%	18% (17/92)	12% (8/67)	1.58 (0.68–3.66)	1.49 (0.62–3.57)※
Kapiga1998 /P. Cohort	Dar es Salaam, Tanzania	Family planning clinic visitors	1022/22	34mons	98%	2.60 per 100 pyo	9.20 per 100 pyo	0.28 (0.09–0.89)	0.29 (0.09–0.97)▽
Reynolds2006 /P. Cohort	Rakai, Uganda	Couples with HIV(+)males	№	№	13%	6.60 per 100 pyo	10.3 per 100 pyo	0.67 (0.45–1.00)	№
Turner2007 /P. Cohort	Zimbabwe	Family planning clinic visitors/STD clinic visitors/sex workers	989/3249	24mons	30%	2.03 per 100 pyo	2.96 per 100 pyo	0.69 (0.48–0.99)	0.78 (0.53–1.14)◇
Baeten2010/P. Cohort	14 sites in east/southern Africa	Couples with HIV(+) males	374/722	24mons	34%	2.72 per 100 pyo	4.38 per 100 pyo	0.62 (0.35–1.10)	0.56 (0.30–1.05)§

RCT: randomised controlled trial. P. Cohort: prospective cohort study.

*Person-years of observation.

#Risk ratio is incidence rate (or hazard) ratios for RCT and Cohort shown.

&Confidence interval.

○Hazard ratio adjusted for sexual behaviour that increased slightly in the intervention group, condom use, and health-seeking behavior.

◎Hazard ratio adjusted for non-adherence to treatment and excluding four men found to be seropositive at enrolment.

⊕Rate ratio adjusted for age, marital status, and sexual risk behaviours at enrolment.

♂Adjusting factors were not achieved.

⊙Rate ratio adjusted for age, marital status, sexual partners in past year, sex for money, condom use and syphilis.

♀Rate ratio adjusted for Hindu/non-Hindu religion, level of education, living with family; and time-dependent covariates: calendar year,age group, marital status, multiple sex partners, number of female sex-worker partners (0, 1, 2–9, or10~), condom use, tattoos, and medical injections.

☆Hazard Ratio adjusted for sociodemographic characteristics at the last trial visit and time-dependent sexual behaviors during posttrial follow up.

※Hazard ratio adjusted for age and condom useage.

▽Rate ratio adjusted for age, marital status, gonorrhea/candidiasis at baseline, number of sexual partners and alcohol consumption during the follow-up period.

◇Hazard ratio adjusted for age, age at coital debut, contraceptive method, husband′s employment status, education, number of partners in past 3 months, and a product-interaction term between time and number of partners in past 3 months.

§Hazard ratio adjusted for male partner HIV-1 plasma viral load and censored at male partner antiretroviral therapy initiation.

№Dates were not available.

According to the recommendations of the Cochrane collaboration, the quality of the 15 studies was assessed based on the study design, conduct, and analysis [23]. Different assessment items were used for RCTs and cohort studies. We evaluated each study using a three-point scale: yes (low risk of bias), no (high risk of bias) and unclear. The heterogeneity of effects between studies was assessed using a χ^2^ test with a significance level of *P* < 0.1. Possible heterogeneity was quantified using the I^2^ statistic (30% < I^2^ < 75%, moderate heterogeneity; I^2^ > 75%, considerable heterogeneity) [24]. Pooled risk ratio(RR) with 95% confidence intervals (CI) was calculated using Mantel-Haenszel random effects model to estimate the effect of male circumcision when I^2^ >75%; otherwise, a fixed effect model was used. Publication bias was assessed using funnel plots [25]. All analyses were performed using Review Manager 5.0.

## Results

### Search results

A total of 4750 citations were identified (**S1 Fig**). Following the patients, intervention, comparison, and outcome (PICO) principle recommend by the Cochrane Collaboration, 4736 were excluded after reviewing the title, abstract, and eventually the full text. Finally, 14 articles (15 studies) were included for meta-analyses. Ten of the 15 studies assessed HIV acquisition for males and 5 analyzed HIV acquisition for females.

### Characteristics of the included studies

One study was a conference abstract [18], and the others were published between 1989 and 2012 (Table 1) [3–11, 19–22]. Of the 15 studies, 4 were RCTs, and the others were prospective cohort studies. The quality evaluation for each study is shown in **S2 Fig**

#### HIV transmission from females to males

Ten studies (three RCTs and seven prospective cohort studies) included 27712 males (circumcised/uncircumcised, 11328/16384). Although Auvert et al enrolled males from the general population initially, only the HIV (−) subpopulation were used for analysis [3]. The subjects in the study by Cameron et al were visitors to an STD clinic; we used only data from the HIV (−) subgroup in the analyses [7]. The remaining eight studies included only HIV (−) males.

#### HIV transmission from males to females

Five studies were related, including one RCT and four prospective cohort studies. Turner et al enrolled females from family planning clinics, STD clinics and sex workers (circumcised/uncircumcised partners, 989/3249) [21], whereas Kapiga et al enrolled only visitors to family planning clinics (circumcised/uncircumcised partners, 1022/22) [20]. Both studies investigated the HIV status of male partners based on the reports of females [20, 21]. Their HIV status were (−) when included, but the HIV status of their male partners was not available. The other three studies contained serodiscordant heterosexual couples, with HIV (−) females and HIV (+) male partners [18, 19, 22].

### Outcome measures

The basic characteristics of all the included studies are shown in Table 1. Eleven authors explicitly calculated the unadjusted and adjusted RRs, whereas four studies (Lavreys et al [17], Wawer et al (s1, s2) [22], and Reynolds et al [20]) only presented the unadjusted RR [8, 11, 19].

#### HIV transmission from females to males

The results of 9 studies assessing HIV transmission from females to males consistently reported a protective effect of circumcision for males themselves. Our pooled analysis for the unadjusted RR was 0.26 (95% CI 0.21–0.32, *P* < 0.00001), and the adjusted RR was 0.30 (95% CI 0.24–0.38, *P* < 0.00001) (**S3 Fig**). The results of sensitivity analysis of the pooled adjusted RR after eliminating 3 studies (Cameron et al, Lavreys et al, and Reynolds et al) were 0.40 (95% CI 0.31–0.53, *P* < 0.00001) [7, 8, 11]. When only 3 RCT studies were considered, the adjusted RR was 0.43 (95% CI 0.29–0.63, *P* < 0.0001).

#### HIV transmission from males to females

The results of the 5 studies assessing HIV transmission from males to females were inconsistent. One RCT showed an insignificant adverse effect, whereas four prospective cohort studies reported a protective effect. The summary unadjusted RR was 0.59 (95%CI 0.40–0.88, *P* = 0.01), and the adjusted RR was 0.55 (95% CI 0.33–0.93, *P* = 0.03) with a moderate heterogeneity (**S4 Fig**). The sensibility analysis result of the pooled adjusted RR was 0.68 (95% CI 0.40–1.15, *P* = 0.15) after excluding studies by Kapiga and Turner [20, 21].

## Discussion

### HIV acquisition for males

Overall, our pooled analysis demonstrated that circumcision provided a 70% protective effect for HIV acquisition. These results were consistent with a previous meta-analysis performed in 2000 [26]. Most studies included in the meta-analysis were cross-sectional or case-controlled studies, without RCTs. They reported a 58% protective effect (adjusted RR: 0.42, 95% CI 0.34–0.54). Here, we only included RCTs or prospective cohort studies. Then, focusing on the 3 excellent RCT studies, they were all stopped early because of ethical concerns for the uncircumcised group [3–5]. Nevertheless, their available data still showed a significant reduction in the incidence of HIV in circumcised males. The study published by Gray et al in 2012 was a prospective post-trial surveillance cohort study that enrolled the study population of a previous RCT performed by the same group in 2007 [5, 12]. This cohort study was performed as an as-treated analysis during a 4.79-year post-trial surveillance, and males were classified by their actual circumcision status. This study provided definitive evidence for the long-term protective effect of male circumcision.

We performed sensitivity analysis by eliminating the studies by Cameron et al, Lavreys et al, and Reynolds et al [7, 8, 11]. The studies by Cameron et al and Lavreys et al both included relatively small population size. Secondly, the full text of the study by Cameron et al hadn’t been acquired. So, it was not available to extract some important information, e.g., follow up duration and HIV (+) rate in both groups. The absence of these data worried us that the duration might be too short to unfold the effect of circumcision. In addition, baseline information in the study by Lavreys et al was not comparable in terms of history of condom use, the use of cigarettes, age, and so on; baseline information on genital ulcers, marital status, and religion differed in the study by Reynolds et al. The conclusion from the newly adjusted RR data was consistent with the previous pooled RR, with a high homogeneity (χ^2^ = 4.66, I^2^ = 0%). This result strengthened our confidence that male circumcision reduced HIV acquisition for circumcised males.

Some of the mechanism underlying the protective effect of circumcision may be related to mucosal immunity and the barrier function of the epithelium. First, males may be easily infected via the inner foreskin and frenulum, which contain large numbers of HIV-targeting Langerhans cells [27]. Their adsorptive capacity is 9-times higher than that of the outer foreskin [28]. In addition, tissue studies have shown that the inner foreskin and frenulum are less keratinized, and that their horny layer is thinner and could easily be scraped. The thinner anatomical barrier between the vaginal secretions at the inner foreskin and the Langerhans cells allow for a more effective penetration of the virus [29]. Male circumcision could sharply decrease the transmission area by removing most of the inner foreskin. Circumcision also reduces the risk of other STDs, which can secondarily reduce the risk of HIV acquisition for males [34–36].

### HIV acquisition for females

Based on the sensibility analysis result (*P* = 0.15), it showed that male circumcision did not provided significant protective effect for their female partners. However, an important issue of the HIV status of women’ partners should be raised.

The 2 cohort studies were conducted among HIV negative women but the HIV status of their partners was not known [20, 21]. However, the studies by the other 3 were conducted in discordant couples. In these studies, HIV incidence was compared in women with an HIV (+) partner who was circumcised with women with an HIV (+) partner who was not circumcised. In other words, the studies in discordant couples should not be mixed up with the studies by Kapiga and Turner because they measure different things. The sensibility analysis result of new pooled RR after excluding the studies by Kapiga and Turner was 0.68 (95% CI 0.40–1.15, *P* = 0.15) and showed a high homogeneity (χ^2^ = 1.02, I^2^ = 0%) [20, 21]. In conclusion, the protective effect of male circumcision to their female partners did not exist based on the current evidence. Our conclusion was consistent with the previous meta-analysis performed by Weiss et al in 2009 [30]. However, they maxed up the different populations as we discussed above; besides, we did not include any cross-sectional studies for the fear of their inherent inferiority to unfold the causality.

After reviewing all the relevant studies included, some issue must be raised. The RCT trial performed by Wawer et al was stopped early by the Data Safety and Management Board for reasons of futility [19]. Consequently, only 92 and 67 serodiscordant couples had been enrolled in to the circumcised and uncircumcised groups, respectively, significantly decreasing the power of the study. The study concluded that circumcision had an adverse effect on HIV acquisition, with an adjusted RR of 1.49 (95% CI 0.62–3.57). A rigorous RCT conducted by Kigozi et al found that the risk of HIV transmission from circumcised HIV (+) males to female partners increased if intercourse occurred before complete wound healing [31]. Consistent with this, Wawer et al demonstrated that, in couples resuming intercourse more than 5 days before male partner’s wound was certified as completely healed and within the 5 days before or any time after certified wound healing, 27.8% (5/18) and 9.5% (6/63) seroconversions were observed (*P* = 0.06), respectively. Therefore, care providers should remind their patients to delay resuming intercourse or use condoms.

The effect of male circumcision on female HIV acquisition may depend on HIV penetration directly from the inner foreskin or frenulum, as well as factors that affect HIV transmission such as genital ulceration and HPV [32, 33]. Published RCTs have reported that male circumcision is associated with a lower incidence of HPV, HSV-2, and syphilis in the circumcised male themselves [34–36]. For example, after a HIV (+) male was circumcised, both the transmission area and the incidence of HPV infection, decreased. Subsequently, his female partner benefited from a reduced transmission area and rate of HPV acquisition, which may lower her risk of HIV acquisition. Unfortunately, our study did not identify any evidence to support this protective effect. Therefore, more convincing data from multicenter RCTs, particularly among serodiscordant couples with HIV (+) males, may reveal a protective effect.

## Limitations

Our study has limitations that must be discussed. All analyses were based on extracted rather than individual patient data; therefore, the results must be interpreted cautiously. Second, publication bias is an additional inherent limitation, since the funnel plot showed evidence of publication bias. Only 2 of 10 studies concerning HIV acquisition for male did not report a protective effect, which may overestimate the effect of male circumcision.

## Conclusions

In conclusion, our results provide strong evidence that male circumcision was effective for reducing HIV acquisition for males. Consequently, WHO and UNAIDS have recommended male circumcision as an important part of a comprehensive HIV prevention package among males [37, 38]. However, no protective effect was found for the female sexual partners of circumcised males. Finally, at high HIV prevalence area, circumcision should be considered as a part of HIV prevention strategies, which should also include HIV counseling and testing, condom promotion, changing sexual habits, and so on. The benefit would then be expanded to its fullest potential for both males and females.

## Supporting Information

S1 PRISMA Checklist (DOC)

S1 Fig Flow diagram for included and excluded articles. (TIF)

S2 Fig Quality evaluation of included studies. *there are 4 RCT. The 6 items on the left side with “?” were used for evaluation of RCT study, and the others 8 items on the right side were used for evaluation of Cohort study.
(TIF)

S3 Fig Forest plot of adjusted RR of HIV acquisition from female to male. (TIF)

S4 Fig Forest plot of adjusted RR of HIV acquisition from male to female. (TIF)
