# Supplementary figures and images for: Circumcision Status and Risk of HIV Acquisition during Heterosexual Intercourse for Both Males and Females: A Meta-Analysis
Source: PLoS One. 2015 May 5;10(5):e0125436. doi: 10.1371/journal.pone.0125436 (PMC4420461; doi:10.1371/journal.pone.0125436)

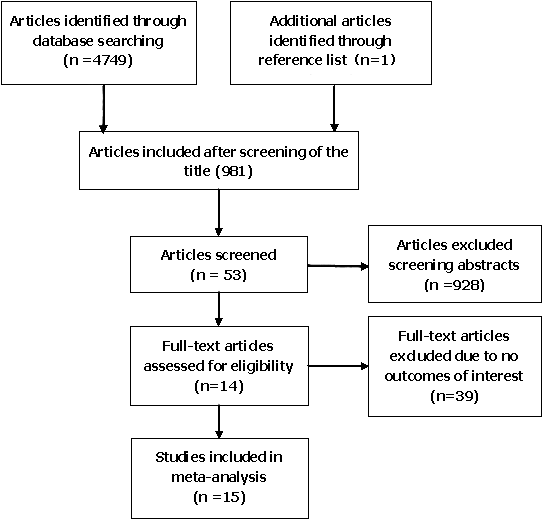

Supplement: S1 Fig — (TIF) [file pone.0125436.s002.tif]

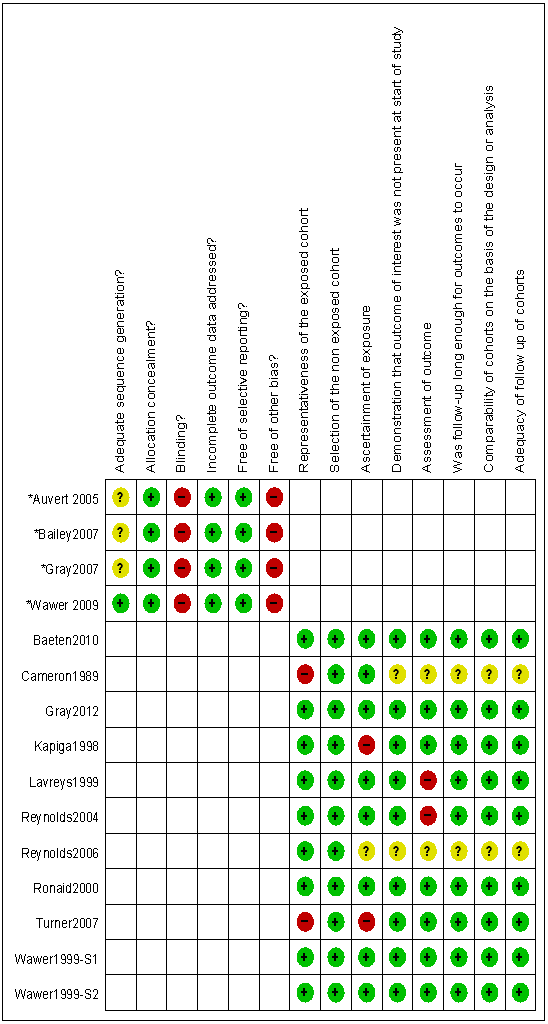

Supplement: S2 Fig — *there are 4 RCT. The 6 items on the left side with “?” were used for evaluation of RCT study, and the others 8 items on the right side were used for evaluation of Cohort study. (TIF) [file pone.0125436.s003.tif]

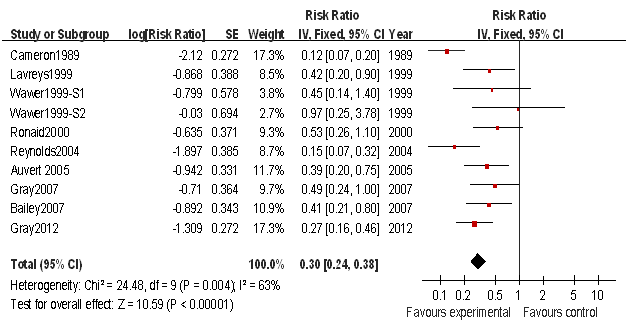

Supplement: S3 Fig — (TIF) [file pone.0125436.s004.tif]

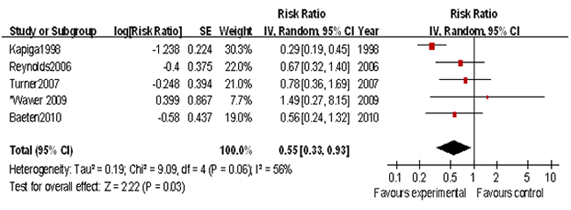

Supplement: S4 Fig — (TIF) [file pone.0125436.s005.tif]
